# The effects of attention on the temporal integration of multisensory stimuli

**DOI:** 10.3389/fnint.2015.00032

**Published:** 2015-04-23

**Authors:** Sarah E. Donohue, Jessica J. Green, Marty G. Woldorff

**Affiliations:** ^1^Center for Cognitive Neuroscience, Duke UniversityDurham, NC, USA; ^2^Department of Neurology, Otto-von-Guericke University MagdeburgMagdeburg, Germany; ^3^Leibniz Institute for NeurobiologyMagdeburg, Germany; ^4^Department of Psychology, University of South CarolinaColumbia, SC, USA; ^5^Department of Psychiatry, Duke UniversityDurham, NC, USA

**Keywords:** attention, multisensory, audiovisual, cueing, bounce-stream, temporal

## Abstract

In unisensory contexts, spatially-focused attention tends to enhance perceptual processing. How attention influences the processing of multisensory stimuli, however, has been of much debate. In some cases, attention has been shown to be important for processes related to the integration of audio-visual stimuli, but in other cases such processes have been reported to occur independently of attention. To address these conflicting results, we performed three experiments to examine how attention interacts with a key facet of multisensory processing: the temporal window of integration (TWI). The first two experiments used a novel cued-spatial-attention version of the bounce/stream illusion, wherein two moving visual stimuli with intersecting paths tend to be perceived as bouncing off rather than streaming through each other when a brief sound occurs near in time. When the task was to report whether the visual stimuli appeared to bounce or stream, attention served to narrow this measure of the TWI and bias perception toward “streaming”. When the participants’ task was to explicitly judge the simultaneity of the sound with the intersection of the moving visual stimuli, however, the results were quite different. Specifically, attention served to mainly widen the TWI, increasing the likelihood of simultaneity perception, while also substantially increasing the simultaneity judgment accuracy when the stimuli were actually physically simultaneous. Finally, in Experiment 3, where the task was to judge the simultaneity of a simple, temporally discrete, flashed visual stimulus and the same brief tone pip, attention had no effect on the measured TWI. These results highlight the flexibility of attention in enhancing multisensory perception and show that the effects of attention on multisensory processing are highly dependent on the task demands and observer goals.

## Introduction

The selective focusing of attention on a particular region in space provides a more accurate representation of the objects that lie within that region than those that lie within unattended regions. With accurate perception being crucial to optimal behavioral responses, the topic of the role that attention plays in enhancing perception has been studied for decades (see Carrasco, 2011 for review). One method that has been used to characterize how attention is focused and the ramifications of that focus is attentional cueing (Posner, 1980). In spatial cueing studies of visual attention, participants are cued to shift their attention to a particular location, while ignoring other locations, in preparation for an upcoming stimulus that is likely to occur in the cued location (e.g., Posner, 1980; Posner and Cohen, 1984; Weichselgartner and Sperling, 1987; Müller and Rabbitt, 1989; Berger et al., 2005; Giordano et al., 2009). When targets appear at the cued (i.e., attended) location, participants are faster and more accurate to respond to them as compared to targets that appear at an uncued location (e.g., Bashinski and Bacharach, 1980; Hawkins et al., 1990; Coull and Nobre, 1998; Yeshurun and Carrasco, 1999). Data from neural studies of the allocation of spatial attention suggest that this improvement in behavioral performance is the result of a gain in the event-related response of the sensory cortices responsible for processing the targets, as well as surrounding inhibition of the processing of the distractors (Motter, 1993; Luck et al., 1994, 1997; Mangun, 1995; Hopf et al., 2006; Silver et al., 2007).

One key feature of attention that has emerged from recent work is the flexibility with which it operates. When the task is to discriminate the orientation of a gabor patch among noise (i.e., to perform a contrast discrimination), the allocation of spatial attention will enhance the signal from that stimulus, enabling enhanced discrimination (Carrasco et al., 2000). When the task is to make fine color discriminations, attention will serve to enhance the processing of the color information (Wegener et al., 2008). In other tasks, attention can serve to enhance relevant information in the face of conflict (e.g., MacDonald et al., 2000; Botvinick et al., 2001), to spread so as to encompass an entire object (e.g., Egly et al., 1994; Donohue et al., 2011), or to aid in the coding of the direction of motion (Stoppel et al., 2011). Although the majority of data on spatial attention has come from studies of the visual modality, auditory and tactile cues can also serve to direct attention to a particular region of space, producing enhanced processing of visual, auditory, or tactile targets that fall within that region (Eimer and Schröger, 1998; Spence et al., 2000; Wu et al., 2007; Green et al., 2011), demonstrating that attention can be flexibly deployed within and across all the spatial modalities.

Perception is not limited to one modality, however, as we can receive spatially-relevant information from visual, auditory, and tactile modalities concurrently. Input from multiple modalities can arise from a multisensory event or object, and this input is often grouped (or integrated) together. The binding of multisensory input occurs when stimuli are temporally and spatially proximal, with the likelihood of such binding falling off as the spatial and/or temporal separation increase (Meredith and Stein, 1986; Meredith et al., 1987; Slutsky and Recanzone, 2001; Zampini et al., 2005; Donohue et al., 2010; reviewed in Chen and Vroomen, 2013). In speech, for example, this binding of multisensory stimuli allows us to associate the auditory (speech sounds) and visual (mouth movements) input as coming from a single individual and not from multiple sources, which facilitates both the perceptual integration of the separate inputs and the accurate processing of the speech information (Besle et al., 2004). That is, when these redundant inputs (i.e., from the same event or object) are grouped, this can facilitate the perceptual processing of that event or object relative to other stimuli in the environment (see Alais et al., 2010 for review).

With both selective attention and multisensory integration generally enhancing the processing of stimuli (e.g., Miller, 1982; Mangun and Hillyard, 1991; Quinlan and Bailey, 1995; Diederich and Colonius, 2004; Pestilli et al., 2007; Abrams et al., 2010; Gondan et al., 2011), it would seem likely that when these two functional processes occur together, the optimal representation of the environment would be obtained. The interaction between selective attention and multisensory integration is not necessarily additive in nature, however, and the degree to which attention and integration are independent processes, and how they interact during perception has been of much debate recently (see Koelewijn et al., 2010; Talsma et al., 2010 for reviews). One example of such a discrepancy comes from studies of perceptual recalibration wherein the perception of audio-visual simultaneity can be altered by exposure to temporal offsets between the auditory and visual stimuli (e.g., Fujisaki et al., 2004). In such cases, the focus of attention appears to be able to influence the audio-visual pairing to which the perception of simultaneity is recalibrated (Heron et al., 2010; Ikumi and Soto-Faraco, 2014). Yet neural evidence from recordings in animals suggests that auditory and visual stimuli can be temporally integrated without attention being necessary (Meredith et al., 1987).

One possible reason for discrepant findings on the degree to which attention and multisensory integration interact is the nature of the specific tasks that have been used. Some studies have employed tasks that require perceptual discriminations in one modality, with the other modality being task-irrelevant (Keitel et al., 2011; Sarmiento et al., 2012; Marchant and Driver, 2013), whereas others have required attention to both modalities, when a target could be present in one or both modalities (e.g., Talsma et al., 2007). Other studies have used tasks that are orthogonal to the question at hand (Busse et al., 2005; Fairhall and Macaluso, 2009), using measurements of neural activity to infer that multisensory integration has taken place or that enhanced processing results from multisensory stimulation. Still others have not required a task at all, assessing “passive” multisensory integration processes (van Atteveldt et al., 2004, 2007). If attention is as flexible a system as research suggests, then it may be the case that under some of these circumstances attention is necessary for effective multisensory integration, whereas in other tasks it may be less essential or have no influence at all (Bertelson et al., 2000).

Here, we focused on one specific facet of multisensory integration—temporal binding—to determine the circumstances under which attention interacts with audiovisual integration processes. As mentioned above, multisensory stimuli tend to be grouped together when they occur close together in time (see Stein and Stanford, 2008 for review). This temporal binding is not absolute, however, and encompasses a window of approximately ±150 ms, known as the temporal window of integration (TWI; Spence et al., 2001; Zampini et al., 2005; van Wassenhove et al., 2007). In general, when an auditory stimulus and a visual stimulus occur within this temporal range they are more likely to be linked together and integrated, whereas with larger temporal separations the stimuli are more likely to be segregated. The breadth of this temporal window, therefore, reflects the temporal precision of the integration process, with a narrow window indicating integration that is in line with physical simultaneity and a broad window indicating integration that occurs relatively far beyond physical simultaneity. Such a temporal spread, therefore, can serve as a useful tool in characterizing the way attention and multisensory integration processes interact.

**Figure 1:**
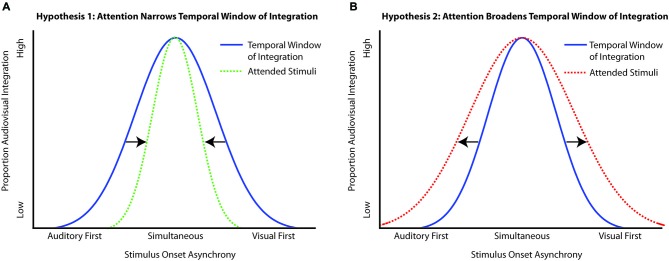
**Possible outcomes of the interaction of attention with the temporal window of integration (TWI). (A)** Hypothesis 1 depicts the narrowing of the TWI under the influence of attention, which would make the integration more precise (i.e., closer in line with the physical offsets of the stimuli). **(B)** Hypothesis 2 depicts the broadening of the TWI when multisensory stimuli are presented in the presence of attention. In this circumstance, attention serves to enhance the integration process overall, thereby producing a greater time range over which the multisensory stimulus components are likely to be integrated.

Utilizing what is known about attention within and across modalities, several possible hypotheses can be generated about the ways in which attention could interact with the TWI. If attention serves to sharpen perception, giving more precision to judgments of what is physically present in the environment, then attention should act to narrow the TWI (Figure 1A). Conversely, because attention is a flexible process, it may be the case that in tasks that are facilitated by multisensory processing, attention will tend to serve to broaden the TWI, making integration more likely over a broader temporal range, thus enhancing the multisensory integration process itself (Figure 1B). Lastly, attention could have no effect on the TWI, with the same amount of temporal integration observed whether stimuli are attended or are unattended.

Here we performed three experiments that used three different tasks and that manipulated the allocation of spatial attention and temporal onsets of the auditory and visual stimuli to determine if attention would sharpen or broaden the TWI. Our results suggest that attention interacts with audiovisual integration processes in a flexible and adaptive manner—broadening the TWI, sharpening the TWI, or not modulating the TWI at all—depending on the requirements of the task and the amount of perceptual uncertainty due to the complexity of the stimuli.

## Experiment 1

In this experiment, we created a cued-attention version of the classic “bounce/stream paradigm” to measure audio-visual integration as a function of attention. In the bounce-stream paradigm, two visual objects (e.g., circular disks) move toward each other, overlap, and then move away from each other (Watanabe and Shimojo, 1998). This pattern of motion can be perceived either as two objects streaming through each other or as two objects bouncing off each other. When the visual objects are presented in this configuration, participants generally tend to perceive them (correctly) as streaming through each other. However, when a sound is presented near the time of overlap, participants are more likely to perceive the objects as bouncing off of each other (Sekuler et al., 1997; Watanabe and Shimojo, 2001; Bushara et al., 2003). That is, although the motion of the visual stimuli is always physically identical, the mere presence of an irrelevant sound can alter the perception of the visual stimuli.

The above-described phenomenon, known as the auditory bounce effect (ABE), has been proposed to be the result of audio-visual integrative processes based on several pieces of evidence. First, the ABE is dependent on the type of sound used, with sounds that are more collision-like in nature producing higher percepts of bouncing (Grassi and Casco, 2009, 2010). Second, when the objects are perceived as bouncing vs. as streaming (under the same physical conditions), multisensory brain regions are activated (Bushara et al., 2003). Third, transcranial magnetic stimulation (TMS) to the right posterior parietal cortex (a region implicated in multisensory processing) decreases the perception of bouncing responses (Maniglia et al., 2012). Finally, the ABE has been shown to decrease as the auditory stimulus is presented farther away in time from being coincident with the visual stimulus (Watanabe and Shimojo, 2001; Remijn et al., 2004), indicating the temporal dependency of this multisensory effect.

In the classic bounce-stream paradigm, the visual stimuli are presented centrally, are the only stimuli presented, and thus occur within the focus of attention. In the current experiment, participants were given an attention-directing cue toward the left or right visual field that indicated where the bouncing/streaming objects were most likely to appear, allowing us to examine responses when the stimuli were occurring within the cued (attended) location vs. when they were occurring within the uncued (unattended) location. In addition, we manipulated the temporal delay between the auditory stimulus and intersection of the visual stimulus pair, as the perception of bouncing should decrease as the temporal discrepancy increases. If attention has no influence on multisensory integration, then we would expect the same pattern of integration-reflecting behavior regardless of whether the auditory and visual events occurred within or outside the focus of attention. If, however, attention interacts with multisensory integration, we would expect a different pattern of perception as a function of attentional allocation. More specifically, we hypothesized that attention would interact with multisensory integration by specifically serving to narrow the TWI, highlighting its ability to provide more accurate representations of objects that fall within its focus (Figure 1A).

## Methods

### Participants

Twenty healthy adults with normal vision and hearing participated in this study (6 male; Mean age = 24.6 years, SD = 4.1 years). One additional participant was excluded due to a failure to understand the task instructions. All procedures were approved by the Institutional Review Board of the Duke University Health System, and all participants gave written informed consent prior to the start of the experiment.

### Stimuli and Task

**Figure 2:**
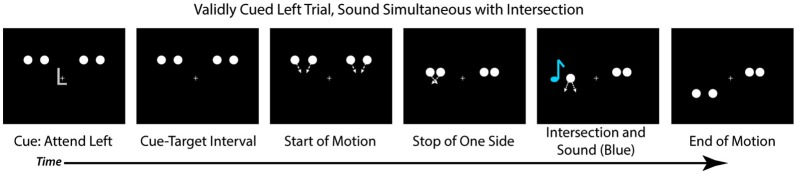
**Trial Structure**. Each trial started with a centrally presented cue (“L” or “R” for left or right), followed by a cue-target delay interval. After the cue-target delay, both sets of disks started moving toward each other. Before they intersected, one set of disks (here depicted on the right) would stop moving while the other would continue to move, intersect and overlap, and move apart, continuing upon the trajectory. In 25% of the trials a sound would occur simultaneously with the disks’ intersection (D0, depicted here), and other trials the sound could be delayed by 150 ms after the intersection (25% of trials; D150), be delayed by 300 ms after the intersection (25% of trials; D300), or not be present at all (25% of trials; VO). Participants were asked to judge if they perceived the disks that continuously moved as bouncing off of each other or streaming through each other.

Each trial began with a central cue at fixation (the letter “L” or “R”) that instructed participants to direct their attention to the left or right hemifield, respectively (Figure 2). At the same time as the cue, four white disks onset bilaterally, two in the upper left hemifield and two in the upper right hemifield. Each disk was 1.5° in diameter and presented 4° above fixation, with the innermost disks 4.9° and the outermost disks 10° to the left and right of fixation. All stimuli were presented on a black background. The cue lasted for 250 ms, after which there was a cue-target interval of 650 ms wherein the disks (and a fixation cross) remained stationary on the screen. After the delay period, each pair of disks began to move toward one another (i.e., the left disks moved toward each other and the right disks moved toward each other). On each trial, one pair of disks continued to move (at a rate of 11 degrees per second), intersected with 100% overlap, and then continued their trajectory until they were 4° below fixation. The pattern of the motion, therefore, was an “X” in shape, and the disks physically always streamed through each other. The second pair of disks stopped moving prior to the intersection such that they never overlapped (See Figure 2 for trial sequence). On 75% of trials the full motion stimulus appeared at the validly cued location and the stopped motion appeared at the uncued location. On the remaining 25% of trials the full motion stimulus appeared at the uncued location, and the stopped motion at the cued location. The next trial began 750 ms after the full motion stimulus ended. Participants were instructed to make a bounce/stream judgment for the full motion stimulus, regardless of the location at which it appeared, and to respond via button press as quickly as possible.

On 25% of trials the visual stimuli were presented alone (Visual Only; VO), allowing us to examine the effects of spatial attention on the perception of the motion stimuli in the absence of any multisensory interactions. On 75% of the trials an auditory stimulus was presented (500 Hz tone, 16 ms duration with 5 ms rise/fall, 50 dB SPL) via speakers positioned adjacent to the computer monitor. On these multisensory trials, the sound could be presented simultaneously with the intersection of the disks (25% of all trials; 0 ms audio-visual delay; [D0]), presented 150 ms after the intersection of the disks (25% of all trials; 150 ms Delay [D150]), or presented 300 ms after the intersection of the disks (25% of all trials; 300 ms Delay [D300]). The sounds were always presented from the speaker on the same side as the full motion visual stimulus to avoid effects of spatial incongruency between the auditory and visual stimuli and increasing the likelihood of multisensory integration (Meredith and Stein, 1986; Slutsky and Recanzone, 2001). Participants were told that some trials would contain a non-informative sound which was not relevant for their responses.

Each participant completed one practice block followed by six experimental blocks. Participants’ eye movements were monitored online via video feed to ensure they were maintaining central fixation. Each block contained 72 valid trials and 24 invalid trials, equally distributed across locations (left/right) and SOAs (D0/D150/D300), for a total of 144 valid and 48 invalid trials for each SOA for each participant. In the VO condition there were also a total of 144 valid and 48 invalid trials presented during the experiment.

### Behavioral Data Analysis

The proportion of “bounce” responses was compared with a repeated-measures ANOVA with factors for validity (2 levels: validly cued targets, invalidly cued targets) and audio-visual delay (3 levels: D0, D150, D300). The VO trials were separately compared for valid vs. invalid cuing with a paired-samples *t*-test. For the response-time data, a 2 × 4 ANOVA was run with the factors of validity (2 levels: validly cued targets, invalidly cued targets) and of condition (4 levels: VO, DO, D150, D300). Greenhouse-Geisser adjusted *p*-values are reported where applicable.

## Results

### Response Times

Prior to performing any analyses of the bounce judgments, response times (RTs) for the valid trials were compared to those for the invalid trials to ensure that the attentional manipulation had been effective. Participants were significantly faster to respond when the target stimuli occurred at the validly cued location as compared to when they occurred at the invalidly cued location (Mean Valid RT = 590 ms, SD = 140; Mean Invalid RT = 666 ms, SD = 140, *F*_(1,19)_ = 28.45, *p* < 0.001, *η_p_*^2^ = 0.60), indicating that the participants were, indeed, attending to the cued side of the display. There was an additional main effect of condition (*F*_(3,57)_ = 15.86, *p* < 0.001, *η_p_*^2^ = 0.46), with the responses to the D0 stimuli being faster than those to the visual alone (Mean D0 = 582 ms, Mean VO = 637 ms, *t*_(19)_ = 4.52, *p* < 0.001), the responses to the D0 condition being faster than the D150 condition (Mean D150 = 647 ms, *t*_(19)_ = 5.15, *p* < 0.001), and the responses to the D0 condition being faster than the responses to the D300 condition (Mean D300 = 646 ms, *t*_(19)_ = 4.46, *p* < 0.001). Of note, all the aforementioned pair-wise comparisons remained significant at the Bonferroni-corrected alpha level of 0.008). For the RTs, there was also a significant interaction between validity and condition (*F*_(3,57)_ = 3.95, *p* = 0.01, *η_p_*^2^ = 0.17), which was driven by the validity effect for the D300 condition being significantly larger than the validity effect for the VO condition (*t*_(19)_ = 3.44, *p* = 0.003).

### Bounce/Stream Judgments

**Figure 3:**
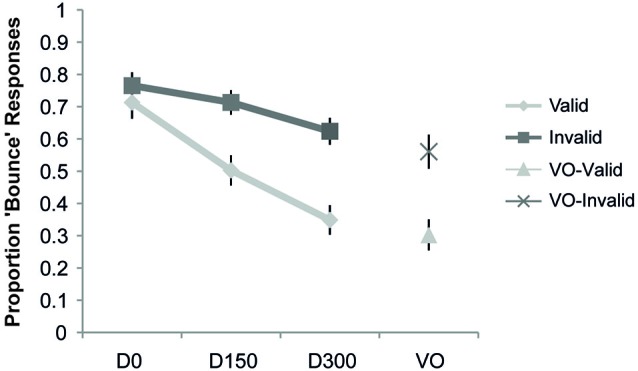
**Results of Experiment 1**. The proportion “bounce” responses are plotted as a function of condition. The respective points represent when there was no auditory stimulus (Visual Only, VO), and when the auditory stimulus occurred simultaneously with the visual intersection (D0), delayed by 150 ms from the visual intersection (D150), and delayed by 300 ms from the visual intersection (D300), separately for the validly and invalidly cued trial types. Compared to the validly cued trials, a significant increase in the bouncing percept was observed in the invalid trials for the VO, 150, and 300 conditions, thus indicating a narrowing of the TWI with attention. Error bars represent the standard error of the mean (SEM).

The proportion “bounce” responses as a function of cue validity is shown in Figure 3. The ANOVA revealed a main effect of validity (*F*_(1,19)_ = 7.98, *p* = 0.01, *η_p_*^2^ = 0.30), a main effect of SOA (*F*_(2,38)_ = 35.46, *p* < 0.001, *η_p_*^2^ = 0.65), and a significant interaction of validity and SOA (*F*_(2,38)_ = 23.99, *p* < 0.001, *η_p_*^2^ = 0.56). *Post hoc* *t*-tests showed a significant difference (at the Bonferroni-corrected alpha of 0.02) between the proportion “bounce” responses for validly and invalidly cued targets at both the 150 ms (*t*_(19)_ = 3.01, *p* = 0.007) and 300 ms delays (*t*_(19)_ = 4.03, *p* = 0.001), with both SOAs showing a higher proportion of “bounce” responses when the stimuli were presented on the invalidly-cued side. In contrast, the simultaneous condition did not reveal any significant differences as a function of cue validity (*t*_(19)_ = 0.87, *p* = 0.40). An analysis of the visual-alone condition also revealed a lower proportion of “bounce” responses for valid compared to invalid trials (*t*_(19)_ = 3.80, *p* = 0.001). Thus, in this experiment the effect of spatial attention was to narrow the TWI, by steepening the roll-off of the SOA function over which the audio-visual information was integrated into a “bouncing” percept (see Figure 3).

## Discussion of Experiment 1

The results of Experiment 1 show that attention does indeed alter the temporal binding of multisensory stimuli. In line with previous studies, the perception of bouncing decreased as the auditory stimulus occurred farther away in time from the visual intersection (Watanabe and Shimojo, 2001; Remijn et al., 2004). This was true for both the validly and invalidly cued trials, and highlights the importance of temporal coincidence in this multisensory percept. However, when the visual and auditory events fell outside of the focus of attention (i.e., occurred at the invalidly cued location), participants were more likely to perceive bouncing when the auditory stimuli were delayed in time. This modulation of perception by attention, even at a delay that is typically considered outside the TWI (300 ms), suggests that the TWI was broadened when attention was not present, or, conversely, that the presence of attention narrowed/steepened the TWI).

One question that arises, however, is whether attention was altering multisensory integration *per se*, or if the effects seen here are driven primarily by attentional modulation of visual perception, as the visual-alone condition showed a similar effect of cue validity. Previous findings have suggested the importance of local motion cues in determining the accurate representation of streaming (Kawabe and Miura, 2006) and that when attention is drawn away from the local motion of the two objects they are more likely to be perceived as bouncing (Watanabe and Shimojo, 1998). Although this visual modulation may be contributing to the effects observed here, it cannot fully account for our results, as the increase in “bounce” perception was not uniform across temporal intervals. The combination of an absence of a validity effect on the bounce perception when the audio-visual stimuli occurred simultaneously and the increasing cue validity effects with increasing temporal disparity suggests that attention interacted with the TWI. Moreover, several recent studies have linked the bounce/stream illusion to multisensory integration by demonstrating that the perception of bouncing is highly dependent on the type of auditory stimuli (with more collision-like stimuli giving a higher proportion of bouncing percepts (Grassi and Casco, 2009, 2010), and that the perception of bouncing both activates multisensory areas in neuroimaging studies and is dependent on the functional integrity of those areas (Bushara et al., 2003; Maniglia et al., 2012).

The pattern observed here is thus consistent with the idea that attention serves to provide the most accurate representation of the information within its focus, whether it be visual alone or visual combined with auditory information. Indeed, participants were more likely to perceive the visual stimulus by itself as streaming (i.e., its veridical physical movement), rather than bouncing, when it was presented inside the focus of attention. Importantly, however, the visual information in this experiment was always attended and the auditory information was always task-irrelevant.

## Experiment 2

Although the results of Experiment 1 provide one way in which the focus of attention can alter the temporal pattern of multisensory integration, the actual judgment of the temporal binding of the visual and auditory stimuli was inferred through a somewhat indirect measure (i.e., the proportion “bounce” responses of the visual stimuli, with only the visual stimuli being relevant). In the second experiment we wanted to more directly assess the temporal binding processes by having both the audio and visual stimuli be task relevant and by making the task entail an explicit judgment of the relative timing of these two stimuli. Accordingly, we asked an independent group of participants to perform a different task using the same stimuli, namely to judge the temporal coincidence of the auditory stimulus and the intersection of the moving circles. We hypothesized that the pattern of temporal integration would be altered in a similar manner as in Experiment 1 such that there would be increased integration at the 150 and 300 ms SOAs when the audio-visual events occurred in an unattended compared to attended visual location (Figure 1A).

### Participants

Twenty participants (9 male) participated in this experiment (Mean age = 22.3 years, SD = 3.2 years). None of the participants in this study had participated in Experiment 1. All participants gave written informed consent and all procedures were approved by the Institutional Review Board of the Duke University Health System.

### Stimuli and Task

The stimuli and experimental conditions were identical to those used in Experiment 1 with the exception of the visual-only condition, which was eliminated due to the task requiring the presence of both the audio and visual stimuli on every trial. In particular, rather than participants judging if the visual disks appeared to bounce or stream through each other, they now performed a simultaneity judgment task. Specifically, participants were asked to determine if the sound occurred at the same time as the intersection of the visual stimuli or if it was offset in time. All responses were made via button press, and participants were instructed to respond as quickly and as accurately as possible. Participants were monitored via a live video feed to ensure they were maintaining fixation.

### Behavioral Data Analysis

Analogous to Experiment 1, the proportion of “simultaneous” responses in the various conditions was compared with a repeated-measures ANOVA, with factors for validity (2 levels: validly cued targets, invalidly cued targets) and audio-visual delay (3 levels: D0, D150, D300). A separate ANOVA with identical factors was conducted for the response time data. Greenhouse-Geisser adjusted p-values are reported where applicable.

## Results

### Response Times

As above, in order to assess the efficacy of the attentional manipulation, RTs to discrimination task for validly cued stimuli were compared to those to invalidly cued stimuli. As in Experiment 1, participants were significantly faster when the multisensory stimuli appeared on the cued (valid) side than on the uncued (invalid) side (Mean valid RT = 649 ms, SD = 162 ms; Mean invalid RT = 704 ms, SD = 188 ms; *F*_(1,19)_ = 11.05, *p* = 0.003, *η_p_*^2^ = 0.36), There was also a main effect of audio-visual delay *F*_(2,40)_ = 77.43, *p* < 0.001, *η_p_*^2^ = 0.80), with participants responding significantly faster (at the Bonferroni-corrected alpha level of 0.017) in the D0 condition (Mean = 589 ms) than in the D150 condition (Mean = 703 ms; *t*_(19)_ = 9.26, *p* < 0.001), as well as faster in the D0 condition than in the D300 condition (Mean = 737 ms; *t*_(19)_ = 912.07, *p* < 0.001). There was also a significant validity by audio-visual delay interaction (*F*_(2,40)_ = 7.36, *p* = 0.003, *η_p_*^2^ = 0.30), which was driven by the validity effect for the D0 condition being greater (at the Bonferroni-corrected alpha level of 0.017) than the validity effect for the D150 condition (*t*_(19)_ = 4.14, *p* = 0.001) and the validity effect for the D0 condition being greater than the validity effect for the D300 condition (*t*_(19)_ = 2.87, *p* = 0.009).

### Simultaneity Judgments

**Figure 4:**
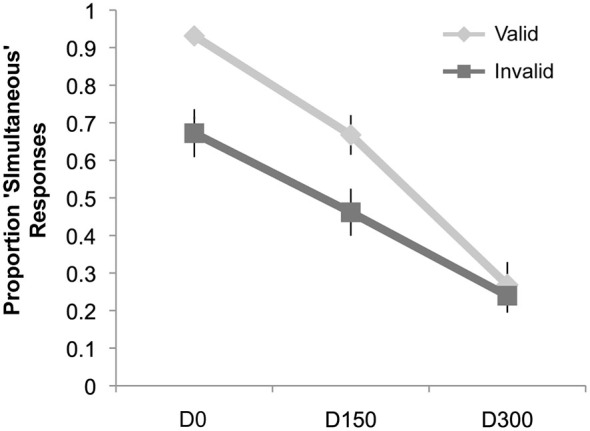
**Results of Experiment 2**. The proportion “simultaneous” responses are plotted as a function of condition: simultaneous auditory stimulus with visual intersection (D0), auditory stimulus delayed by 150 ms from the visual intersection (D150), and the auditory stimulus delayed by 300 ms (D300), separately for the validly and invalidly cued trial types. There was a significant decrease in the proportion of “simultaneous” responses for the D0 and D150 conditions when these trial types were presented on the invalidly cued side (compared to the validly cued side), but no difference for the D300 condition.

Results of the simultaneity judgment task can be seen in Figure 4. Similar to Experiment 1, we observed a main effect of validity (*F*_(1,19)_ = 10.46, *p* = 0.004, *η_p_*^2^ = 0.36), a main effect of SOA (*F*_(2,38)_ = 68.23, *p* < 0.001, *η_p_*^2^ = 0.78), and an interaction of validity and SOA (*F*_(2,38)_ = 11.40, *p* < 0.001, *η_p_*^2^ = 0.38). However, *post hoc* *t*-tests (significant at the Bonferroni-corrected alpha level of 0.02) revealed that there were significantly fewer “simultaneous” responses for invalidly cued trials at both the D0 (Mean Valid = 93.1%, Mean Invalid = 67.2%; *t*_(19)_ = 4.02, *p* = 0.001) and 150 ms delay (Mean Valid = 66.8%, Mean Invalid = 46.2%; *t*_(19)_ = 3.15, *p* = 0.005). No cue validity effect was observed at the longest SOA.

## Discussion of Experiment 2

In this second experiment, now using a simultaneity judgment task, attention also interacted robustly with the temporal pattern of multisensory integration; however, this interaction manifested in a completely different manner than in Experiment 1. Whereas in Experiment 1 the measure for integration (a “bounce” perception) was *more* likely to occur at the unattended location, at least as the multisensory components were more separated in time, here the measure for integration (a simultaneity judgment) was *less* likely to occur at the unattended location, particularly when the audio and visual events were closer in time even physically simultaneous and would have been expected to be temporally integrated. This discrepancy indicates that the specifics of the task, such as whether only one or both modalities are attended or whether the temporal relationship of the stimuli is task relevant, can dramatically influence the interaction of attention and stimulus binding or integration processes.

One important difference between the two above experiments was the task-relevance of the auditory stimuli. The audiovisual stimuli were physically identical in both experiments, but in Experiment 1 only the visual stimuli were task-relevant whereas in Experiment 2 information about both the auditory and visual events was necessary for responding correctly. Thus, the simultaneity judgment task may have imparted an increased level of uncertainty about the stimuli, or at least an increased level of complexity of the task: Not only did participants need to determine when the visual stimuli intersected, but they also needed to decide if the auditory stimulus coincided with this intersection. Having attention directed away from the uncued side may have increased the uncertainty of the timing of both the visual intersection and that of the auditory stimulus, while also increasing the complexity of the task, which may have resulted in the shift in criterion that we see here. In other words, if both the intersection of the disks and the auditory tone were happening outside the focus of attention, the greater temporal ambiguity of the two relevant events may have caused participants to be less likely to link them in time (as measured by their judgment of the simultaneity).

A second interpretation of the pattern of results observed here is that when, as in Experiment 2, the task specifically required the discrimination of the temporal relationship of the stimuli and thus the explicit discrimination of the temporal binding, attention served to increase the precision of such binding by integrating events that were temporally concurrent (i.e., in the simultaneous condition), while still segregating appropriately those events that were temporally separate (i.e., the D300 condition). However, when the measure of multisensory integration is more direct as in the bouncing/streaming task of Experiment 1 (although it is perhaps a more indirect measure of temporal linking), attention served to provide the most precise representation of the visual motion, with more veridical streaming percepts when the stimuli occurred within its focus.

A slightly different interpretation of these results could be one of a shift in criterion. When the auditory and visual stimuli were presented at the attended location, it could be the case that participants shifted their criteria toward judging the stimuli as being simultaneous. In other words, with the exception of the widest SOA (D300), participants were more prone to responding “simultaneous” when attention was present. Again, given that this effect did not occur for every SOA, it was not independent of the timing, but rather served to increase the “simultaneous” responses particularly for the D0 and D150 conditions. From such an interpretation, it would follow that attention serves to enhance the temporal binding of auditory-visual stimuli, making them more likely to be perceived as being temporally simultaneous, and tending to create more certainty for making a “simultaneous” response.

Overall these first two experiments both clearly show that attention can strongly modulate the processes related to the temporal integration of multisensory stimuli. Although the measures of temporal integration and segregation differed for the two tasks, in both Experiments 1 and 2 there was a clear modulation of widely used measures of integration by attention. The strikingly different patterns of this attentional modulation depended strongly on the measures being used and on what task was being performed. More fundamentally, the results suggest that the role of attention in multisensory integration processes can change depending on the task being performed with the sensory stimuli, in a way that is in line with the idea that attention serves to resolve ambiguity in our perception, depending on what type of information is important for veridical perception. It also suggests that the temporal binding of multisensory stimuli (as measured with the simultaneity judgment task) differs from the perceptual integration of multisensory stimuli (measured in the bounce/stream task).

Both the bounce/stream task and the simultaneity judgment task used here required precise tracking of moving visual stimuli over one second, with successful performance depending on determining what was occurring during one specific moment of this motion (i.e., the stimulus intersection). The disruption of this trajectory when participants had to switch to the uncued side likely increased the uncertainty of the disk intersection timing, as well as the nature of this perceptual event. Not only do these complex stimuli increase perceptual uncertainty overall when they occur on the unattended side, the long-duration motion stimuli are quite different than the static, highly-controlled, flashed visual stimuli often used in multisensory integration tasks (Spence et al., 2001; Zampini et al., 2005; Donohue et al., 2010) Thus, it remained unclear what role attention would play in audiovisual integration under situations with little-to-no perceptual uncertainty, such as with discrete temporal events. Accordingly, we further wanted to ascertain how specific this modulation was to the stimuli that we had been using, and therefore conducted a cued-variant of a simultaneity judgment task using temporally discrete stimuli in both the visual and auditory modalities.

## Experiment 3

Experiment 3 utilized the same task as in Experiment 2 (a cued simultaneity-judgment task) but replaced the complex visual motion stimuli with a flash of a static checkerboard image. We hypothesized that this simple task, involving a temporally discrete visual stimulus and a temporally discrete auditory one, would involve less perceptual uncertainty and less task complexity, and thus that the influence of attention on the TWI would be reduced, as attentional resources would be less essential for accurate task performance.

### Participants

Twenty participants took part in this experiment (one left-handed, nine male, Mean age = 23.6, SD = 4.5 years). None of these participants had taken part in either of the previous experiments. Three additional participants were excluded for failure to do the task properly (i.e., having less than 50% accuracy for reporting the simultaneous trials as simultaneous on the validly cued side). All participants gave written informed consent, and all procedures were approved by the Institutional Review Board of the Duke University Health System.

### Stimuli and Task

**Figure 5:**
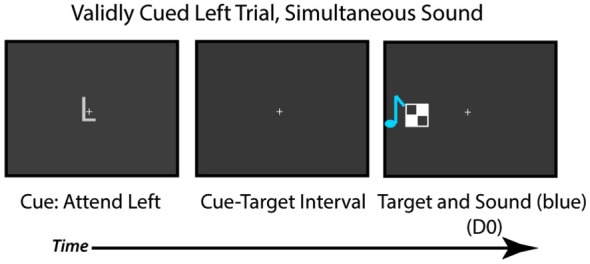
**Task used in Experiment 3**. Participants were cued to attend to the left or right visual field. After a cue-target interval, the checkerboard target appeared on the validly cued (75% of time) or invalidly-cued side (25% of the time), and participants had to judge if the sound and visual target occurred simultaneously or at separate times.

The auditory stimuli used were the same as in Experiment 2 (brief tone pips), but the visual motion stimuli were replaced by a briefly flashed (16 ms duration) static black and white checkerboard (1.8° × 1.8°) that could be presented 12.6° to the left or right of fixation and presented on a gray background (Figure 5). In addition, on each trial, the checkerboards were only presented on one side of fixation (as opposed to the bilateral displays used in the previous experiments). The checkerboard image was presented on the validy cued side 75% of the time and on the invalidly cued side 25% of the time). There were 18 trials in each block for each of the valid conditions and 6 trials in each block for each of the invalidly-cued conditions, with six blocks in total completed by each participant.

As in Experiment 2, participants performed a simultaneity judgment of the audiovisual events. Participants were asked to determine if the checkerboard image and the tone occurred at the same time or if they occurred at slightly different times, and to respond as quickly and as accurately as possible via button press. As with all experiments, participants were monitored over a closed-circuit video camera to ensure they were maintaining fixation and remaining attentive to the task.

### Data Analysis

All data analyses of the simultaneity judgment’s task measures were performed in an identical manner to Experiment 2.

## Results

### Response Time

Participants were significantly faster to respond to the validly cued trials compared to the invalidly cued trials (Mean valid RT = 630 ms, SD = 60 ms; Mean invalid RT = 651 ms, SD = 69 ms; (*F*_(1,19)_ = 12.15, *p* = 0.002, *η_p_*^2^ = 0.39), demonstrating participants were focusing their attention toward the cued location. There was also a main effect of audio-visual SOA (SOA (*F*_(2,38)_ = 109.46, *p* < 0.001, *η_p_*^2^ = 0.85) with all conditions differing significantly from each other at the Bonferroni-corrected alpha level of 0.0167 (Mean RT D0 = 560 ms, Mean RT D150 = 640 ms, Mean RT D300 = 720 ms, all *p*’s < 0.001). The interaction between validity and audio-visual significance did not reach significance (*F* < 1).

### Simultaneity Judgments

**Figure 6:**
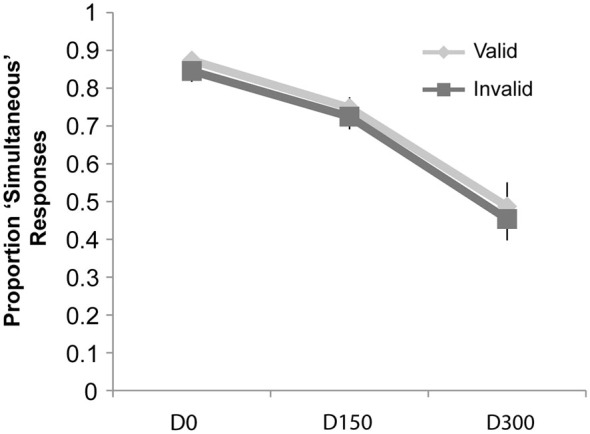
**Proportion “Simultaneous” responses for the cued simultaneity judgment task of Experiment 3**. While there was a main effect of SOA, no effects of attention or an interaction of attention and validity were observed.

The repeated-measures ANOVA on the proportion of “simultaneous” responses (see Figure 6) revealed a main effect of SOA (*F*_(2,38)_ = 36.74, *p* < 0.001, *η_p_*^2^ = 0.66), but no significant effect of validity nor a significant interaction of validity and SOA (all *p*’s > 0.05).

## Discussion of Experiment 3

In Experiment 3, although the proportion of “simultaneous” responses showed the classic decrease with the temporal separation of the visual and auditory stimuli, there were no differences observed at any of the SOAs as a function of attention. Thus, with the use of simple, briefly presented stimuli in both modalities, attention did not influence the TWI. Importantly, there was still a validity effect on the RTs, suggesting that participants were appropriately focusing their attention to the cued location (as in Yeshurun and Carrasco, 1999).

These data suggest that attention is not necessary for processes related to multisensory integration in all cases. Indeed, much of the pioneering work on the neural basis of multisensory integration was done with recordings in the superior colliculus of anesthetized cats (Meredith and Stein, 1986; Meredith et al., 1987). In this case, the animals were not paying attention to the simple audiovisual stimuli (flashes and tones) and yet the temporal and spatial properties of multisensory integration were still observed (Meredith and Stein, 1986; Meredith et al., 1987).

More likely, because the stimuli in Experiment 3 were simple, discrete perceptual events, without the added ambiguity or complexity of predicting motion trajectories, attention was not needed to help resolve the perceptual uncertainty of their timing. The brief flash of a checkerboard image on the screen provides a temporally isolated event, whereas in the case of the bounce/stream stimuli, the intersection of the two disks is part of a continuously moving image. Moreover, the checkerboard was only presented on one side of the screen, reducing any location uncertainty and thus also perhaps increasing binding between the single auditory and single visual stimulus. In contrast, in Experiments 1 and 2 there was always an incomplete motion stimulus on the opposite side of the screen of the full motion target. This may have reduced the ability or speed for participants to shift their attention to the invalidly cued location in response to the onset of motion, as desired for those paradigms, while also adding to the perceptual complexity of the task.

## General Discussion

In a series of experiments, we observed three different behavioral patterns that reflect ways in which attention can interact with the TWI for audiovisual stimuli. In the first experiment, when the relative stimulus timing was not relevant for the participants’ task, attention served to narrow the TWI. In the second experiment, when the task involved explicitly judging the audiovisual synchrony, attention served mainly to broaden the TWI, making the SOA function both wider and steeper. In the third experiment, also requiring explicit judgment of temporal synchrony but when simple, unambiguous, briefly presented stimuli were used in both the visual and auditory modalities, attention had no effect on the measured TWI. Although at first glance it would appear that these findings are contradictory, each result provides a piece of evidence that together demonstrate the adaptable nature of attention. Specifically, when attention acts in the context of multisensory stimuli it is able to do so in a flexible manner, enhancing that which is most relevant for a given task.

In Experiment 1, the visual stimuli were configured such that they could be perceived to bounce off of each other or stream through each other. Thus, perceptual ambiguity was created by both the local motion of the visual stimuli over a period of time, with the intersection occurring very briefly within that period, and by the presence of task-irrelevant yet perceptually influential auditory stimuli. In order to be successful at this task, attention must be implemented in such a way as to enhance accurate perception of the visual motion. When the intersection occurred on the unattended side, the participants likely did not have as clear a representation of the motion trajectory because they had not been attending to that side in the first place, and some information about the motion trajectory may have been lost in the switch of attention to the unattended side. Additionally, attention could help by segregating or suppressing the irrelevant auditory stimulus so that it was not perceived as part of the multisensory event. In line with this interpretation, we observed fewer reports of “bouncing” when the auditory stimulus was offset in time from the visual event and attention was present, thereby narrowing the TWI, as proposed in the potential outcomes in Figure 1A. Further, in the unimodal (visual only) condition, having attention present also helped participants achieve the veridical perception of streaming, confirming previous findings of the importance of attention to local motion cues in the bounce/stream paradigm (Watanabe and Shimojo, 1998).

Despite the use of identical stimuli in Experiment 2, the effects of attention were quite different from those in Experiment 1. Simply switching the task from a bounce/stream judgment of the visual stimuli, where the sounds were an irrelevant distraction, to a simultaneity judgment, where the sounds were necessary for task performance, resulted in a very different pattern of effects of attention on the TWI function. With the simultaneity judgment task, attention served to cause some broadening of the temporal window by increasing the amount of temporal disparity that the multisensory inputs could have and still result in a perception of simultaneity, particularly in the D150 condition. However, the results were not completely in line with the simple “broadening” process shown in Figure 1B. The greatest effect here was at the SOA of 0 (i.e., where the stimulus events were actually simultaneous), in that attention served to increase the number of “simultaneous” responses reported (from ~68% for unattended to ~93% for attended—see Figure 4). Notably, this D0 effect also meant that the TWI was actually both broadened *and* steepened by attention in this experiment.

Importantly, the perceptual challenge of the task in Experiment 2 was to determine if the auditory stimulus occurred at the precise moment where the moving visual stimuli intersected. For this to be successfully done, the participant had to focus on the motion trajectory of stimuli while preparing for the auditory stimulus. Thus, the large D0 effect indicates that attention served to enhance perception in the simultaneous condition by appropriately grouping the auditory and visual events together. It is somewhat less clear why attention would serve to increase the proportion of “Simultaneous” responses in the D150 condition; one possibility, however, is that anticipatory attention builds up as the visual motion nears the point of intersection, enhancing audio-visual integration, and then tapers off after the intersection has occurred. The continued enhancement for the 150 ms delay would therefore be merely a by-product of the enhanced integration that was temporally aligned with the visual intersection that had not yet had time to dissipate.

The results of the first two experiments, together, suggest that when auditory and visual stimuli are presented such that multiple interpretations of what physically occurred are possible, attention aids in resolving this ambiguity in a flexible manner depending on task demands. These findings demonstrate that attention can act in multisensory contexts much as it does in unimodal contexts: by helping the processing of that which is relevant for the most successful, appropriate behavior. Additionally, just as in unimodal contexts, attention here did not appear to have a strict gating mechanism; that is, there was still some processing of stimuli that was the same in the presence or absence of attention (the D0 condition in Experiment 1 and the D300 condition in Experiment 2). Such low-level interactions support previous research that has suggested that multisensory integration can occur without the presence of attention (Vroomen et al., 2001), and these interactions can still fall off as the temporal separation between the stimuli increases (Meredith et al., 1987).

In Experiment 3, however, when simple, temporally discrete, brief stimuli were used in both modalities, attention appeared to have no effect on the temporal integration and segregation of multisensory stimuli. As most of the previous work on multisensory temporal processing in both humans (Spence et al., 2001; Zampini et al., 2005; Donohue et al., 2010, 2011) and animals (Meredith et al., 1987) has used simple stimuli, it is not surprising that robust multisensory interactions have still been found neurally in the absence of attention (Meredith et al., 1987). Critically, here we show that, under circumstances with very simple brief stimuli, with no motion trajectories needed to calculate, attention did not seem to be needed for, or to show any influence on, temporal coincidence judgments. It is also the case that the discrete onsets/offsets in Experiment 3 of both the auditory and the visual stimuli were more likely able to exogenously capture attention such that the judgments of the relative timing could be accomplished even when attention was not initially in place at the spatial location of the stimuli. Indeed, the difference in reaction times between the attended and unattended sides was smallest in Experiment 3, suggesting that attention was indeed more easily and rapidly shifted to the unattended side in this case, and this likely influenced the pattern of behavior we observed here. Moreover, the overall RTs were faster in the simultaneity judgment task in Experiment 3 as compared to that in Experiment 2, further suggesting that the task itself was overall easier, consistent with the view that attention would not need to play as important a role there.

Another important issue to consider here is whether the multisensory stimuli were actually being perceptually integrated in all of these experiments, or if other aspects of multisensory processing were being measured which do not necessarily involve perceptual integration. In the bounce/stream task used here in Experiment 1, the task involves reporting the perception of visual stimuli, namely whether the perception of two crossing visual stimuli is perceived as crossing or bouncing, and this perception varies as a function of the presentation of an irrelevant auditory stimulus. This influence by the auditory stimulus on the perception of the visual stimuli would thus appear to accurately reflect the perceptual integration of these multisensory inputs, and these effects have been previously interpreted in this way. Moreover, previous research has provided converging evidence that the integration of audio-visual information underlies the enhanced perception of bouncing in the bounce/stream task by demonstrating the involvement of multisensory regions in the “bounce” percept (Bushara et al., 2003; Maniglia et al., 2012) and the role that the attributes of the sound stimuli play in these judgments (Grassi and Casco, 2009, 2010). Accordingly, the bounce/stream task would seem to be is a fitting direct measure of multisensory integration (although perhaps a somewhat indirect measure of temporal linking).

Simultaneity judgment paradigms, on the other hand, where the explicit task is to judge the temporal relationship between two events in different modalities, are a less direct measure of multisensory integration *per se*. Although there is strong evidence to suggest that when stimuli are temporally coincident, or are perceived as temporally coincident, they will tend to be integrated into a single event (see Stein and Stanford, 2008 for review), it is possible that auditory and visual stimuli could be judged as occurring at the same time without actually being perceptually integrated, just as two visual stimuli could be judged as occurring simultaneously even though they are perceived as separate and discrete perceptual events. Without other evidence to support the actual occurrence of multisensory integration in the simultaneity judgment experiments, another explanation for the discrepant findings here is that attention may play an entirely different role when two multisensory stimuli are being perceptually integrated vs. when they just temporally interact or their temporal relationship is being discriminated.

Within the multisensory integration literature, there has been some evidence to suggest that temporal binding and the perceptual integration of multisensory stimuli are different processes. Such an example can be found in the McGurk illusion, wherein a subjects is presented with a face mouthing the sound “ga” and while hearing the sound “ba”, resulting in the perception of “da” (McGurk and MacDonald, 1976). In a series of studies using this illusion, (Soto-Faraco and Alsius, 2007, 2009) it was found that when participants were presented with these stimuli at various SOAs and asked to report both their percept and if the auditory and visual stimuli occurred simultaneously (or the temporal order of the stimuli), they observed that these two types of judgments did not necessarily line up. At some SOAs participants correctly identified the temporal order of the stimuli (or perceived them as temporally asynchronous) despite still perceiving the McGurk illusion, suggesting the stimuli were fused into one multisensory percept. Of course, these processes are not completely independent, given that temporal binding may often be necessary for auditory and visual events to be integrated (a moving mouth and voice would not be associated as coming from the same source if they were substantially temporally offset). Nevertheless, these previous results provide evidence that simultaneity judgment tasks (or temporal-order judgment tasks) do not always produce judgments of the temporal aspects of stimuli that directly correspond to perceptual integration of the exact same stimuli.

Moreover, the present results indicate more generally that the notion of multisensory integration vs. temporal binding is a key point that requires further research. Although it would seem to be the case that temporal binding is necessary for multisensory integration to occur, it may not be sufficient for such a process, in that it would seem that events could be temporally bound or linked without being perceptually integrated into a multisensory object. If these two processes were always identical, then the way in which attention should interact with them should also be identical; our current results, however, clearly indicate otherwise. Thus, although tasks such as simultaneity judgment tasks may be useful to assess the temporal linking of stimuli, they are likely not tapping into the multisensory integration processes in the same way that a bounce/stream task does.

Although attention modulated the functional patterns of our measures of the TWI, the absence of attention did not eliminate integration or binding. Across all experiments, integration still followed a temporal gradient, with more temporally coincident stimuli being more likely to be integrated and more temporally disparate stimuli tending to be segregated. It is not surprising, however, that some perceptual information is still getting through even on the unattended side. Classic ERP studies of visual attention show that although attention serves to enhance a sensory-evoked response to a visual stimulus, it is not that the visual stimuli in the unattended location do not evoke any neural response at all; in fact unattended stimuli clearly evoke sensory responses, albeit smaller (e.g., Voorhis and Hillyard, 1977). Thus, multisensory integration processes can occur in the absence of attention, and may tend to be fairly accurate, especially for simple, more unambiguous stimuli. With increasingly complex and ambiguous stimuli, however, attention would appear to enhance our ability to successfully integrate or segregate auditory and visual inputs as required by the task at hand. As such, our findings are not consistent with there being only one mechanism of the influence of attention on multisensory processing (Figure 1), but rather would appear to indicate a more complex, dynamic process.

Our pattern of results is consistent with prior work on the interactions of attention and multisensory integration, with some important expansion of previous findings. First, the simultaneity judgment task of Experiment 3, which showed that attention did not modulate the TWI is consistent with some studies in humans suggesting that attention does not modulate the ventriloquism effect (Bertelson et al., 2000) and with studies in animals showing integration in anesthetized cats (e.g., Meredith et al., 1987). Second, the stimuli which contained a motion aspect in the present study (Experiments 1 and 2) showed that attention can modulate the binding of multisensory stimuli, consistent with other studies showing that attention can modulate the BOLD response to congruent audio-visual speech (Fairhall and Macaluso, 2009), can modulate multisensory integration at early perceptual stages (~80 ms; Talsma and Woldorff, 2005), and can enhance EEG components associated with the perception of a multisensory “extra-flash” illusion (Mishra et al., 2010). Together, many of these studies have found enhancements of a neural response to multisensory stimuli in the presence of attention, and our behavioral data extend these findings suggesting that what is “enhanced” can be highly dependent on the goals and needs of the task.

Based on these results, the hypotheses put forth in the introduction (Figure 1) can be reformulated to reflect broader principles concerning multisensory processing and integration. One overarching principle is that the influence of attention on the multisensory TWI is flexible and can adapt to the quality and complexity of the incoming information and to the perceiver’s task needs. Relatedly, the influence of attention on the window of integration also depends on whether the measures of that window reflect true multisensory perceptual integration or reflect simply a temporal linking process. Thus, when only one modality is relevant for a task, as in Experiment 1 for example, attention will tend to narrow the temporal window over which input from an irrelevant modality will be perceptually integrated. In contrast (Experiment 2), when the task involves judging if two stimuli from the different modalities are simultaneous (and thus both are relevant), attention appears to broaden the window of integration, while also making physically simultaneous stimuli more likely to be viewed as such, thereby also resulting in a taller center of the integration window. Another major principle of the influence of attention on multisensory processing is that it tends to decrease as the quality of the sensory input increases, such that in simpler, less ambiguous situations the interactions between attention and multisensory integration processes will be smaller. Thus, with very simple, discrete stimuli, as in Experiment 3, attention will tend to not alter the perception of temporal relationships between them, likely because perception is already more than sufficient and doesn’t require attentional enhancement. More generally, the influence of attention on multisensory processing will vary depending on the task-relevance of the stimulus information from the different modalities, the nature and complexity of those stimuli, and the specific task goals.

Our results suggest that multisensory integration, at least temporal judgments of integration, can sometimes be a bottom-up process operating largely independently from attention, but it can also be substantially modulated by top-down attentional goals, particularly in situations with more complex sensory input or task needs. We propose that the interactions between attention and multisensory stimuli should not be thought of as a single process operating the same way in all cases, but rather as being context- and task-dependent, providing perceptual enhancements of multisensory stimuli as needed to maximize perception and performance.

## Conflict of Interest Statement

The authors declare that the research was conducted in the absence of any commercial or financial relationships that could be construed as a potential conflict of interest.
